# Case Report: Bigeminy with Alternating Injury Pattern Morphologies in a Young Woman After Cardiac Arrest

**DOI:** 10.5811/cpcem.38069

**Published:** 2025-08-18

**Authors:** Madelyn Huttner, Mitchell McMurray, Martin Huecker, Sohail Ikram

**Affiliations:** University of Louisville, School of Medicine, Department of Emergency Medicine, Louisville, Kentucky

**Keywords:** electrocardiogram, myocardial infarction, bigeminy, discordance

## Abstract

**Introduction:**

Coronary artery disease is uncommon in adults under the age of 35, and studies show a lower incidence in women of this age group. Physicians should suspect myocardial infarction in all patients who present with cardiac arrest and a shockable rhythm.

**Case Report:**

We report a case of a 34-year-old female who presented after return of spontaneous circulation following both pulseless electrical activity and ventricular fibrillation. The initial emergency department 12-lead electrocardiogram (ECG) demonstrated ST-segment elevation in the anterior precordial leads. The second, more notable, ECG showed a unique ischemic pattern of ventricular bigeminy with each beat containing a different morphology of injury pattern. Emergent cardiac catheterization found a 100% occlusion of the proximal left anterior descending artery.

**Conclusion:**

Premature ventricular (or junctional) contractions can indicate ischemia when the morphology consists of excessive discordance between the QRS complex and the ST segment and T wave. This case illustrates the importance of scrutinizing each beat in every lead to increase sensitivity for ischemia.

## INTRODUCTION

Coronary artery disease is uncommon in adults under age 35, and studies show an even lower incidence in women of this age group.^1^ Myocardial infarction (MI) should be suspected in patients with prehospital ventricular fibrillation. The standard of care for patients with return of spontaneous circulation (ROSC) is to have electrocardiograms (ECG) performed as early as possible, understanding that false-positive ST-segment elevation myocardial infarction (STEMI) can occur in ECGs obtained within eight minutes of ROSC.^2^

## CASE REPORT

A 34-year-old female with a past medical history of type 2 diabetes, depression, anxiety, obesity, and tobacco use presented to the emergency department (ED) in cardiac arrest. Per emergency medical services (EMS), the patient had chest pain for one day and then collapsed in front of family. They found the patient in pulseless electrical activity (PEA) that transitioned to ventricular fibrillation. En route, the EMS team defibrillated the patient twice and administered the following medications: 300 milligrams (mg) amiodarone; six mg total epinephrine, and two mg naloxone.

**Image 1:**
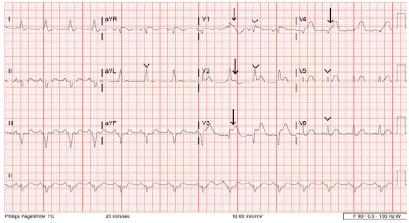
Electrocardiogram demonstrates ST-segment segment elevation in V1–V4 (arrows) with widened QRS complex in aVL, V1, V2, V5, and V6 (arrowheads) in a 34-yr-old female patient after cardiac arrest.

Upon arrival to the ED the patient was intubated and noted to be in PEA. In the ED another 1 mg of epinephrine, one ampule of sodium bicarbonate, and two grams of magnesium sulfate intravenous were administered along with continued chest compressions. After >30 minutes without a pulse, ROSC was obtained, and the initial ECG obtained five minutes post-ROSC demonstrated ST-segment elevations in leads V1-V3 (Image 1). Lead V4 demonstrated hyperacute T waves. The ECG also showed a widened QRS complex with prominent T waves in aVL, V1, V2, V5 and V6. The precordial lead had an inconsistent R-wave progression, with R’ wave present in V1, no R wave (a QS wave) in V3, and no R wave (but a hyperacute T wave) in V4. Limb leads showed subtle ST-segment depression in II, III, and aVF that likely represented reciprocal changes.

An additional 150 mg amiodarone was administered for intermittent/non-sustained ventricular tachycardia. A chest radiograph demonstrated bilateral pulmonary edema, and a point-of-care cardiac ultrasound showed reduced ejection fraction. The medical intensive care unit and cardiology teams were consulted. Due to severe acidosis and hypoxemia, the cardiology team recommended medical stabilization before taking the patient emergently to cardiac catheterization.

**Image 2:**
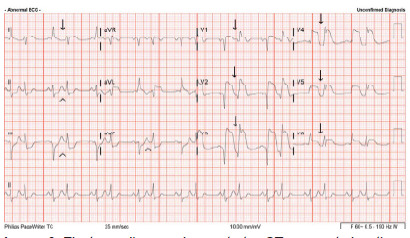
Electrocardiogram demonstrates ST-segment elevation in V1–V6 and lead I with alternating QRS-complex morphologies (arrows) and reciprocal ST-segment depressions in leads II, III, aVF (arrowheads).

The repeat ECG showed bigeminy, ventricular rate of 124 beats per minute, with the unique injury pattern (Image 2). Each of the bigeminy beats were consistent with infarction but in different morphologies. The first beat showed anterolateral ST-segment elevation with QS waves in V1–V3. The second bigeminy beat showed apparent R waves in anteroseptal leads with a deep notch at the J point. It is unclear whether the beats represented premature ventricular contractions or junctional beats. For instance, the first of the two beats appeared to have a P wave preceding the QRS complex. The inferior limb leads showed reciprocal ST-segment depressions, but again with different morphologies.

*CPC-EM Capsule*

What do we already know about this clinical entity?

*False positive ST-segment elevation can occur in electrocardiograms obtained within eight minutes of return of spontaneous circulation (ROSC)*.

What makes this presentation of disease reportable?

*The second ECG shows a unique ischemic pattern of ventricular bigeminy with each beat containing a different morphology of injury pattern*.

How might this improve emergency medicine practice?

*It is important to scrutinize each beat in every lead for ischemia and to evaluate for the persistence of post-ROSC ECG abnormalities with a repeat ECG in 10–20 minutes*.

The initial troponin level resulted at 131 nanograms per liter (ng/L) (reference range: 0–19 ng/L). Rectal aspirin was administered, and the patient was taken for emergent cardiac catheterization.

Coronary angiogram revealed a 100% occlusion of the proximal-mid segment of the left anterior descending artery. The occlusion was treated with a 3.5 × 28 millimeter Xience Skypoint stent (Abbott Laboratories, Abbott Park, IL). A repeat ECG after cardiac catheterization demonstrated sinus tachycardia with left anterior fascicular block and improved ST-segment elevations in V1–V3. The patient remained ventilated, on a heparin drip, and on vasopressors. On hospital day 8, she died peacefully with family at bedside.

## DISCUSSION

Coronary artery disease is rare in adults under the age of 35.^1^ An observational study evaluating young adults with STEMI found common risk factors of male sex, hypertension, and obesity.^1^ Patients in this age group may experience a delay in diagnosis due to low suspicion for ischemia.^3^ Observational data suggest that women are less likely than men to receive ECGs or fibrinolysis within benchmark timeframes (and less likely to receive percutaneous coronary intervention in general).^4^ Risk factors for MI in young female patients include stress, anxiety, and depression.^5^

Patients presenting in cardiac arrest with ventricular fibrillation should be presumed to have MI until proven otherwise. Electrocardiograms obtained within eight minutes of ROSC may show a pattern injury in the absence of coronary occlusion.^2^ Serial ECGs can help determine the persistence of concerning abnormalities as the cardiac membrane stabilizes. The initial ECG in this case led to concern for acute MI, with clear indication of active ischemia. Understanding that early post-ROSC ECGs can mislead, the ED obtained a second ECG (Image 2) and found the unique patterns of ischemia. Bundle branch blocks and paced rhythms can present challenges to detecting ischemia.

**Image 3:**
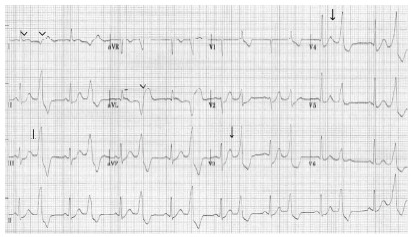
Electrocardiogram demonstrates premature ventricular complexes in a bigeminy pattern with ST-segment depression in differing morphologies in multiple leads (arrows), along with ST-segment elevation in leads I and avL (arrowheads).

Our literature search found no published case with an alternating pattern of injury (Image 2). The first beat showed anterolateral ST-segment elevation, suggesting vessel occlusion. The second beat displays R waves in anteroseptal leads with a deep notch at the J point. As shown in Image 3 from an open-access ECG database, typical premature ventricular complexes (PVC) have a widened QRS complex but usually have an appropriately discordant ST-segment and T wave (Image 3).^7^

## CONCLUSION

Physicians should scrutinize the 12-lead ECGs looking closely at the morphology of all beats (including premature ventricular complexes and bundle branch blocks) to detect ischemia. To evaluate for the persistence of post-ROSC ECG abnormalities, physicians should obtain a repeat ECG within 10–20 minutes.
